# Selenoprotein W expression and regulation in mouse brain and neurons

**DOI:** 10.1002/brb3.159

**Published:** 2013-08-02

**Authors:** Arjun V Raman, Matthew W Pitts, Ali Seyedali, Ann C Hashimoto, Frederick P Bellinger, Marla J Berry

**Affiliations:** Cell and Molecular Biology Department John A. Burns School of Medicine, University of Hawai'i at ManoaHonolulu, Hawaii

**Keywords:** Selenium, selenocysteine, selenoprotein, selenoprotein P, selenoprotein W

## Abstract

**Background** Selenoprotein W (Sepw1) is a selenium-containing protein that is abundant in brain and muscle of vertebrate animals. Muscular expression of Sepw1 is reduced by dietary selenium (Se) deficiency in mammals, whereas brain expression is maintained. However, expression of Sepw1 depends on the Se transporter selenoprotein P (Sepp1). **Methods** We assessed the regional and cellular expression of Sepw1 in the mouse brain and neuronal cultures. **Results** We found that Sepw1 is widespread in neurons and neuropil of mouse brain and appears in both the soma and processes of neurons in culture. Pyramidal neurons of cortex and hippocampus express high levels of Sepw1. It is also abundant in Purkinje neurons and their dendritic arbors in the cerebellum. Analysis of synaptosome fractions prepared from mice brains indicated that Sepw1 is present at synapses, as were several proteins involved in selenoprotein synthesis. Synaptic expression of Sepw1 expression is reduced in mice lacking Sepp1 compared with control mice, although selenoprotein synthesis factors were similarly expressed in both genotypes. Lastly, Sepw1 mRNA coimmunoprecipitates with Staufen 2 protein in a human neuronal cell line. **Conclusions** Our results suggest that Sepw1 may be locally synthesized in distal compartments of neurons including synapses.

## Introduction

Selenium (Se) is an essential micronutrient that is incorporated into antioxidant enzymes. Se is unique among trace elements because it is covalently incorporated into proteins as a noncanonical amino acid, selenocysteine (Sec). Biosynthesis and integration of Sec in polypeptides is distinctive because the residue is specified by a UGA codon, which is typically a stop codon. Recoding the UGA codon for Sec incorporation requires an mRNA that contains a Sec insertion sequence (SECIS), a tRNA that recognizes UGA, and several specific proteins beyond the conventional translation machinery (reviewed in Bellinger et al. 2009). The 25 primate, and 24 murine, Sec-containing selenoproteins identified to date include the functionally characterized glutathione peroxidase (GPX), thioredoxin reductase, and iodothyronine deiodinase enzyme families.

Selenoprotein W (Sepw1) is the smallest mammalian selenoprotein and is one of the most widely distributed selenoproteins across species in all domains of life (Zhang and Gladyshev 2008; Lobanov et al. 2009). Sepw1 was initially identified by its absence in muscle of myopathic lambs suffering White Muscle disease, and was later purified and cloned (Vendeland et al. 1993, 1995; Whanger 2000). White muscle disease is a Se-responsive muscular dystrophy syndrome in sheep and cattle that is characterized by pale and dry muscle, with longitudinal striations or chalky whiteness due to abnormal calcium deposition. Leg muscles typically degenerate first, but all muscles, including cardiac, can be affected.

In addition to muscle and proliferating myoblasts, mammalian Sepw1 is highly expressed in the developing and adult brain (Gu et al. 2000; Loflin et al. 2006). Unlike in muscle, dietary Se deficiency does not reduce Sepw1 levels in sheep or rat brain, despite reducing brain Se concentration and GPX activity (Sun et al. 2001; Whanger 2001). Selenoprotein P (Sepp1) maintains stable selenoprotein expression in the brain under Se deficiency, and mice lacking Sepp1 have greatly reduced levels of Sepw1 mRNA and protein in the brain (Hoffmann et al. 2007). Feeding a Se-deficient diet to mice lacking Sec lyase (Scly) also reduces Sepw1 protein in the brain (Raman et al. 2012). These results suggest that preferential retention of Sepw1 in brain during dietary Se deficiency is maintained by Sepp1 and Scly. Regional analysis of Sepw1 mRNA expression in the brains of mice indicates presence in neurons, with high expression in >90% of brain regions (Zhang et al. 2008). However, the expression, regulation, and function of Sepw1 in the brain are largely unknown.

Like most selenoproteins, Sepw1 is expected to be involved in oxidation–reduction (redox) reactions. It has been shown to act as a glutathione-dependent antioxidant that protects cells from peroxide-mediated damage (Jeong et al. 2002). However, the specific antioxidant function of Sepw1 has been disputed (Xiao-Long et al. 2010), and a prominent role in cell signaling has also been proposed (Hawkes and Alkan 2010). Sepw1 directly interacts with the beta and gamma isoforms of 14-3-3 proteins (Aachmann et al. 2007; Dikiy et al. 2007). Further, siRNA knockdown of Sepw1 expression halts cell cycle progression and inhibits epithelial cell proliferation via a p53- and p21-dependent mechanism (Hawkes and Alkan 2011; Hawkes et al. 2012). Cell cycle arrest at the G1 stage caused by Sepw1 knockdown is mediated by MKK4 and downstream MAPK signaling (Hawkes and Alkan 2012). Sepw1 has also been implicated in cell cycle recovery from G2 arrest induced by DNA damage (Park et al. 2012).

In this report, we analyzed expression of Sepw1 in mouse brain-derived primary neurons and mouse brain. We report that Sepw1 protein expression is observed in neurons of several mouse brain regions including cortex, hippocampus, and cerebellum. Sepw1 immunoreactivity extends into the processes of these cells, and isolation of nerve terminals by synaptosome preparations revealed the presence of Sepw1. We have also identified components of the selenoprotein synthesis machinery in synaptosomes. Additionally, expression of Sepw1 in synaptosomes was reduced in Sepp1-deficient mice, despite no change in selenoprotein synthesis machinery. Finally, we found Sepw1 mRNA in Staufen 2-immunoprecipitated samples from human SH-SY5Y neuroblastoma cells. Taken together these data indicate that Sepw1 is widely expressed in neurons and synapses, and suggest translational regulation of Sepw1 by the RNA-binding protein Staufen 2.

## Material and Methods

### Cell culture

Glass bottom tissue culture plates (World Precision Instruments, Sarasota, FL) were coated with 0.1 mg/mL laminin in 0.1 mg/mL poly-l-lysine solution for 1 h, and then rinsed with phosphate-buffered saline (PBS). Primary cells from cortex, hippocampus, and cerebellum were harvested from postnatal day one C57BL/6 mice, gently dispersed by trituration, and plated on coated dishes. Cultures were maintained at 37°C with 5% CO_2_ and 5% relative humidity in Neurobasal-A medium (Life Technologies, Carlsbad, CA) supplemented with 5% fetal bovine serum (FBS) and 100 μmol/L glutamate (Life Technologies) to reduce growth of glia and enrich growth of neurons. B27 supplement (Life Technologies) was added to replace FBS after 24 h, and glutamate omitted from the media after 3 days. The SH-SY5Y human neuroblastoma cell line was plated in Dulbecco's modified Eagle medium supplemented with 10% FBS. After 24 h the media was switched to Neurobasal medium supplemented with B27 to differentiate the cells. Primary cells were imaged and SH-SY5Y cells were harvested after 2 weeks in vitro. FBS lots were tested for Se content (Bodycote, Santa Fe Springs, CA), and the Se concentration of media containing 10% FBS was 105 nmol/L as determined using inductively coupled plasma-mass spectrometry. B27 was tested for Se content (Bodycote) and the Se concentration of media containing 2% B27 was 93.8 nmol/L by the same method.

### Animals

C57BL/6 mice and genetically modified male mice on a C57BL/6 background lacking Sepp1 were bred on commercially available diets containing adequate Se (∼0.25 ppm). Animals were given food and water ad libitum on a 12-hour light cycle and group housed until experimentation. All experiments were conducted on adult mice aged 3–4 months during the light cycle. All animal procedures and experimental protocols were approved by the University of Hawaii Institutional Animal Care and Use Committee.

### Tissue preparation

Mice were anesthetized with ketamine-xylazine, and sacrificed by transcardial perfusion. Mice were initially perfused with PBS to flush out blood, followed by perfusion with 4% paraformaldehyde (PFA) to fix the tissue. The mice heads were cut off and the brains dissected out. Brains were postfixed in 4% PFA overnight, followed by cryoprotecting the tissue in 10% and 30% sucrose for at least 4 h each. The brains were then embedded in optimal cutting temperature (OCT) compound and frozen until time of sectioning. Forty micrometer sections were cut on a Leica CM1900 cryostat and saved in cryopreservative solution, containing 0.1 mol/L phosphate buffer, 30% sucrose (w/v), and 30% ethylene glycol (v/v) at −20°C, until further experimentation.

### Immunohistochemistry

Primary cortical, hippocampal, and cerebellar cultures maintained for 3 weeks in vitro were used for immunolabeling. Brain sections stored in cryopreservative in the freezer were warmed to room temperature, and sections containing cortex, hippocampus, and cerebellum were selected for analysis. After thorough washing, sections were blocked in 5% normal goat serum with 0.3% Triton X-100 in PBS. After blocking, the sections were incubated in diluted primary antibody solution overnight at 4°C. The following antibodies were used: Rabbit-anti-Sepw1 (Rockland, Gilbertsville, PA) and Mouse-anti-Tuj1 (Covance, Honolulu, HI). A control section where primary antibody was omitted was also included in the procedure. After washing out primary antibody, sections were incubated in species-matched secondary antibody. The secondary antibody was directly conjugated to fluorophores (Alexa Fluor dyes, Life Technologies) for fluorescence imaging. Additionally, some sections were dual labeled with a fluorescent Nissl stain to label neurons (Neurotrace, Life Technologies). Sections were then mounted onto slides, and coverslipped in VectaShield containing DAPI for fluorescent labeling of nuclei. Additional sections were colorimetrically developed using 3,3′-diaminobenzidene (DAB), after signal amplification using the avidin-biotin complex method (Vector Labs, Burlingame, CA), and coverslipped using Permount.

### Synaptosome preparation

Synaptosomes were prepared by the method of Dunkley et al. (1986). Mice were anesthetized with tribromoethanol and sacrificed by decapitation. The brain was rapidly excised, rinsed in ice-cold 0.32 mol/L sucrose, and immersed in ice-cold 0.32 mol/L sucrose with 1 mmol/L ethylenediaminetetraacetic acid (EDTA). Brain tissue was homogenized in 5 mL of ice-cold sucrose/EDTA by 10 strokes at 900 rpm using a prechilled Teflon/glass homogenizer. The homogenate was centrifuged at 3600 rpm for 10 min at 4°C in polycarbonate tubes. The resulting supernatant was collected and diluted with sucrose/EDTA to a total volume of 9 mL. Approximately 3 mL of diluted supernatant was loaded on the top of a discontinuous three layer Percoll gradient. Three gradients per brain were made by adding 2 mL of 23% Percoll to each polycarbonate tube, and slowly layering 2 mL each of 10% and 3% Percoll sequentially using a peristaltic pump. The gradients with sample were centrifuged at 20,000 rpm for 5 min at 4°C to isolate synaptosomes. Isolated synaptosomes were collected from the interface band between the 23% and 10% Percoll layers in each gradient, and transferred and pooled directly to a large polycarbonate centrifuge tube. To wash synaptosomes, 25 mL of HEPES-buffered saline (HBS) was added to the tube, and was centrifuged at 15,000 rpm for 10 min at 4°C. The pellet was resuspended in HBS, and centrifuged at 7000 rpm for 7 min at 4°C. The final pellet was resuspended in HBS for analysis by sodium dodecyl sulfate polyacrylamide gel electrophoresis (SDS-PAGE) followed by western blotting to select selenoproteins and related factors.

### SDS-PAGE and Western blot

Total protein was extracted from S1 fractions by light sonication in CelLytic MT buffer (Sigma, St. Louis, MO) containing dithiothreitol, EDTA, and protease inhibitors, followed by centrifugation according to the manufacturers' protocol. Synaptosomes were resuspended in CelLytic MT buffer without sonication or centrifugation. Protein was added to reduced Laemmli buffer, boiled for 10 min, and loaded into 4–20% gradient polyacrylamide gels (Bio-Rad, Hercules, CA). Following electrophoresis, gel contents were transferred to Polyvinylidene fluoride membranes, which were blocked with undiluted Odyssey Blocking Buffer (Li-Cor Biosciences, Lincoln, NE) for 1 h. Membranes were then probed for 90 min with the following primary antibodies: Rabbit-anti-GPX4 (AbFrontier, Seoul, Korea), Rabbit-anti-SEPW1 (Rockland), Rabbit-anti-SEPHS2 (Rockland), Rabbit-anti-SecP43 (Santa Cruz Biotech, Santa Cruz, CA), Goat-anti-SBP2 (Everest Biotech, Oxfordshire, U.K.), Rabbit-anti-EFSec (AbCam, Cambridge, MA), Mouse-anti-TBP (AbCam), Mouse-anti-beta-actin (Sigma, St. Louis, MO), and Mouse-anti-alpha Tubulin (Novus, Littleton, CO). Rabbit polyclonal Scly antiserum was a kind gift from Dr. Suguru Kurokawa, and has been previously described (Kurokawa et al. 2011; Seale et al. 2012). After washing with PBS containing 0.05% tween-20 (PBST), membranes were incubated in the dark in secondary antibodies labeled with infrared fluorophores (Li-Cor Biosciences). After further washes in PBST, blots were imaged and quantified with the Odyssey infrared imaging system (Li-Cor Biosciences).

### RNA immunoprecipitation and real-time quantitative PCR

STAU1 or STAU2 antibodies (MBL International, Woburn, MA) were conjugated to magnetic DynaBeads (Life Technologies) according to the manufacturer's instructions. Differentiated SH-SY5Y cells were gently harvested by adding chilled lysis buffer containing protease and RNAse inhibitors to the cultures for 5 min. The lysate was collected and an aliquot was saved for analysis of total RNA, while the remainder was incubated with antibody-conjugated DynaBeads for 1 h at 4°C. The beads were then washed and spiked with synthetic RNA as an internal control. RNA was simultaneously isolated from the immunoprecipitated samples and total lysate aliquots by Trizol-chloroform extraction. The concentration and purity of RNA were determined using an ND1000 spectrophotometer (NanoDrop Technologies, Wilmington, DE). Synthesis of cDNA was carried out using High Capacity cDNA Reverse Transcription Kit (Applied Biosystems, Foster City, CA), with 1 μg of RNA per 20-μL reaction. For real-time quantitative PCR (RT-qPCR), 100 ng of the cDNA was used in 5-μL reactions with PerfeCTa SYBR Green FastMix (Quanta Biosciences, Gaithersburg, MD). Reactions were carried out in triplicate in a LightCycler 480 II thermal cycler (Roche, Indianapolis, IN). Cycling conditions followed the manufacturer's suggestions in the SYBR Green kit instructions. All qPCR results were normalized to the spiked synthetic RNA or endogenous hypoxanthine-guanine phosphoribosyltransferase (HPRT) mRNA expression and analyzed using Absolute Quantification Software (Roche).

### Imaging

Fluorescence imaging of primary cultures and stained sections were performed on a Zeiss LSM 5 Pascal laser confocal inverted microscope equipped Ar and HeNe lasers. AlexaFluor-488, -546, and -633 secondary antibodies with directly conjugated fluorophores were used to detect primary antibody signals. Images were acquired using the included LSM software, and were analyzed using ImageJ. Bright-field imaging was performed using an upright Zeiss AxioScope 2 Plus microscope equipped with an ASI motorized stage and Zeiss Axiocam MRc camera.

### Statistical analysis

Data were analyzed using Microsoft Excel (Redmond, WA), and plotted using GraphPad Prism software (San Diego, CA). Unpaired *t*-tests were performed for protein expression in synaptosome experiments and mRNA expression in RT-qPCR experiments. The significance criteria were set at *P* < 0.05 for statistical measures.

## Results

**Figure 1:**
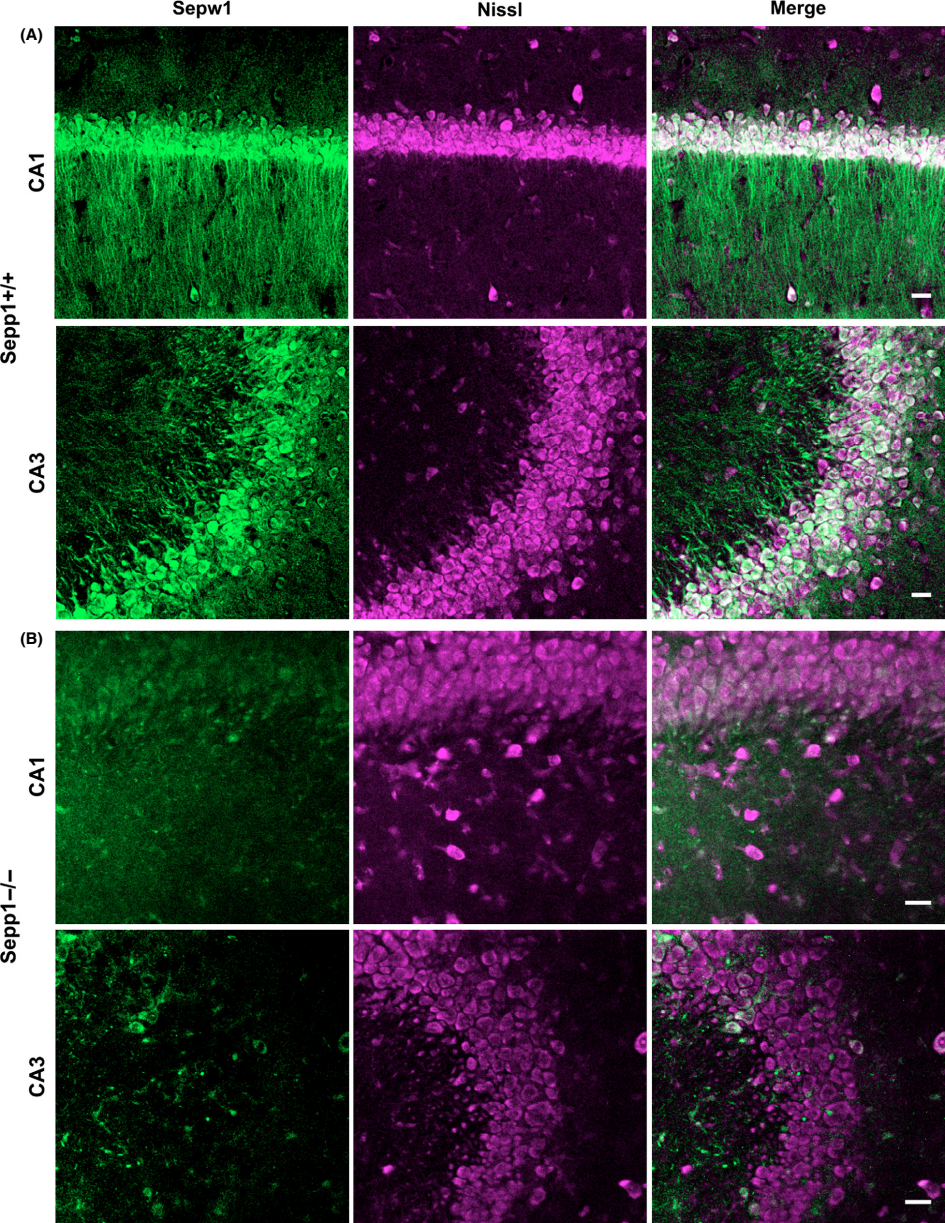
Expression of Sepw1 in cell bodies and processes of pyramidal neurons in hippocampus is reduced in Sepp1−/− mice. Brain sections containing hippocampus were immunolabeled for Sepw1 and combined with a fluorescent Nissl stain in wild-type Sepp1+/+ mice (A) and Sepp1−/− mice (B). Large pyramidal neurons in both CA1 and CA3 displayed Sepw1 immunoreactivity, which prominently extended into the apical dendrites of Sepp1+/+ mice. Sepp1−/− mice had drastically reduced immunoreactivity toward Sepw1. Scale bar = 20 microns.

Selenoprotein W is abundant in the mammalian brain and its mRNA is found in mouse brain neurons; however, the cellular location of the protein has not been described. We sought to determine whether Sepw1 is expressed in neurons of mouse brain, and if neuronal expression of Sepw1 is reduced in Sepp1−/− mice. To address this question, we performed dual label fluorescent imaging of fixed mouse brain sections containing hippocampus using Sepw1 antibodies and a fluorescent Nissl stain to label neurons. In CA1 and CA3 regions of wild-type mouse hippocampus, we observed robust Sepw1 expression in pyramidal neurons (Fig. 1A). Sepw1 expression extended into the apical dendrites of most pyramidal neurons, and was apparent in distal dendritic compartments as well. However, in hippocampus of Sepp1−/− mice (Fig. 1B), the pyramidal layer showed very little immunolabeling of Sepw1 in CA1 or CA3. These data indicate that hippocampal pyramidal neurons are dependent on Sepp1 for Sepw1 expression.

**Figure 2:**
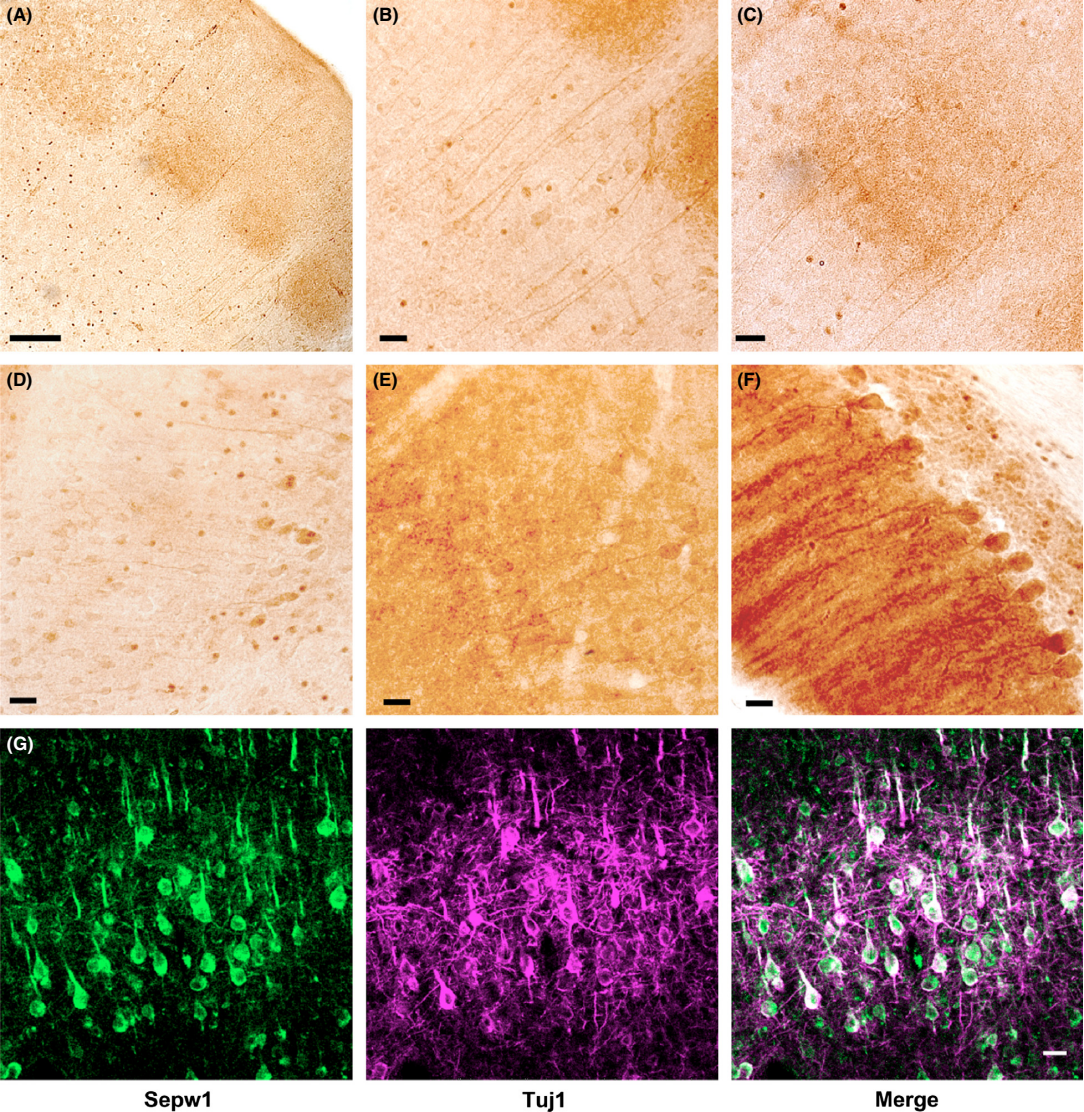
Regional expression of Selenoprotein W (Sepw1) in neurons of mouse brain. (A) Barrel field of somatosensory cortex displayed Sepw1 staining in cell bodies, which extended into processes (B), and was visible in barrels (C). Photomicrographs of cingulate cortex (D), piriform cortex (E), and cerebellum (F) show Sepw1 expression in neurons. Pyramidal neurons and Purkinje neurons display high Sepw1 immunoreactivity in apical dendrites. (G) Imaging of Sepw1 (green, left) and Tuj1 (magenta, center) in somatosensory cortex demonstrates neuronal localization of Sepw1 and some colocalization (white, right) with Tuj1. Scale bar = 100 microns in A; Scale bar = 20 microns in B–G.

To further analyze regional expression of Sepw1, we performed immunohistochemistry on wild-type mice brains. Extending the previous results in hippocampus, Sepw1 expression was observed in somas and apical dendrites of somatosensory cortex barrel field neurons (Fig. 2A–B). Additionally, Sepw1 expression was strong in the barrels (Fig. 2C). Cingulate cortex (Fig. 2D) and piriform cortex (Fig. 2E) displayed high Sepw1 immunoreactivity in pyramidal neurons. Purkinje neurons of cerebellum (Fig. 2F), and their heavily branched dendritic arbors, also showed abundant expression of Sepw1. In fact, most neurons appeared to express Sepw1 and neuropil generally appeared immunopositive for Sepw1. Conspicuously, large neurons showed immunoreactivity extending into the processes. To confirm the Sepw1 staining was in neuronal processes, we stained cortical sections for Sepw1 and the neuron-specific class III beta-tubulin (Tuj1). Sepw1 immunoreactivity was observed in Tuj1-positive cells in somatosensory cortex of mice brains, appearing in neuronal perikarya and proximal dendrites (Fig. 2G).

**Figure 3:**
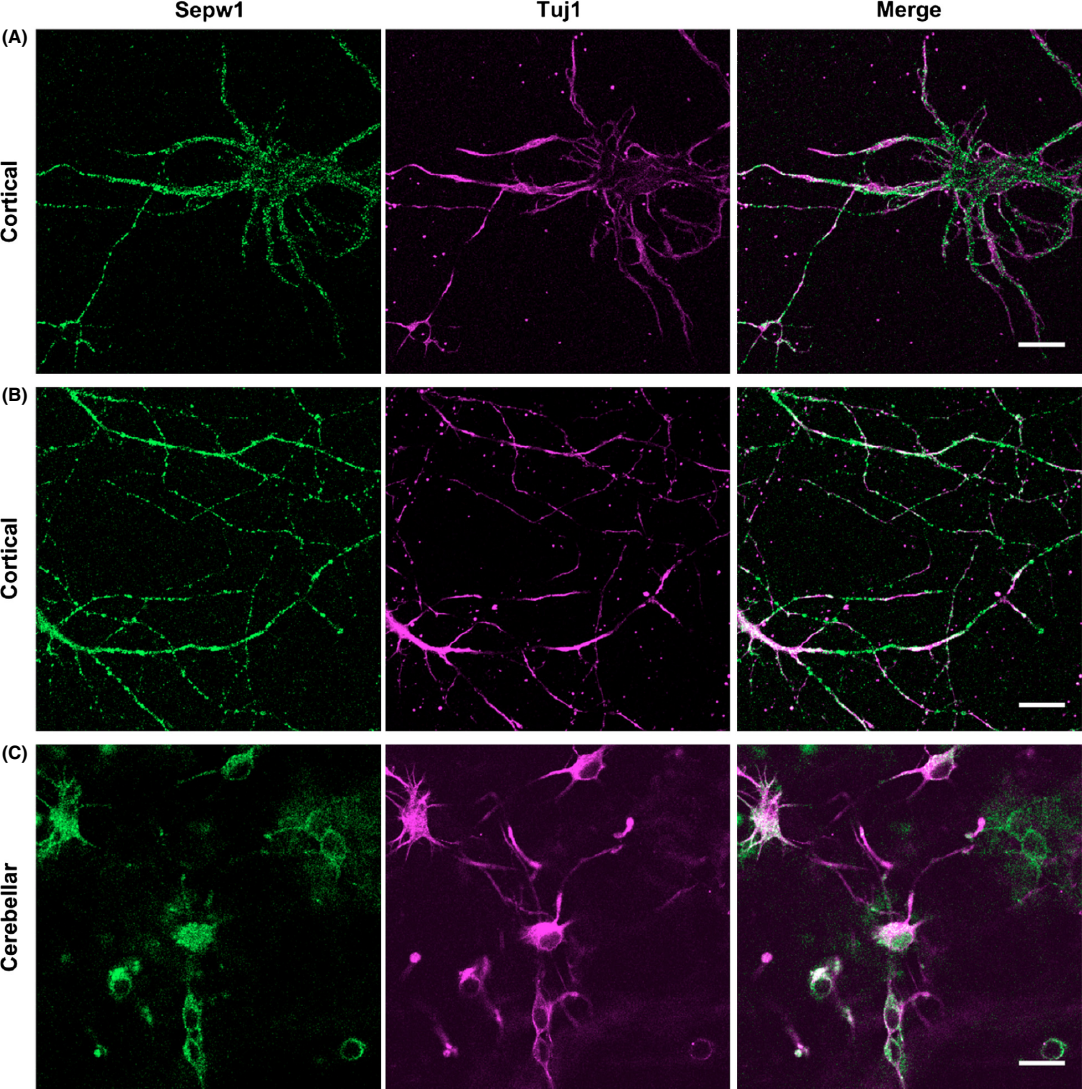
Selenoprotein W (Sepw1) is expressed in cell bodies and processes of neurons in culture. Primary cultures derived from neonatal mouse cortex (A) and (B), and cerebellum (C) were grown on coverslips for 3 weeks and subsequently double immunolabeled for Sepw1 (green, left) and a neuron-specific tubulin, Tuj1 (magenta, center). Merged images show sporadic colocalization between Sepw1 and Tuj1 (white, right) in neuronal soma and neurites in cultures. Scale bar = 20 microns.

Widespread Sepw1 expression in neurons and dendritic processes of adult mouse brain promoted further investigation using cultured neurons. Cultured primary cells harvested from neonatal mouse brain were assessed for expression of Sepw1 along with Tuj1. Primary cultures consisted mainly of neurons, and Tuj1 immunoreactivity (magenta, middle) showed some overlap with Sepw1 expression (green, left) in neuronal cell bodies and neurites. Primary neuronal cultures derived from neonatal cortex (Fig. 3A and B) and cerebellum (Fig. 3C) displayed robust Sepw1 expression, and some colocalization with Tuj1, as indicated by white color in the merged panels (Fig. 3, right). Punctate Sepw1 labeling was observed in neuronal processes (Fig. 3B), suggesting a possible synaptic localization.

**Figure 4:**
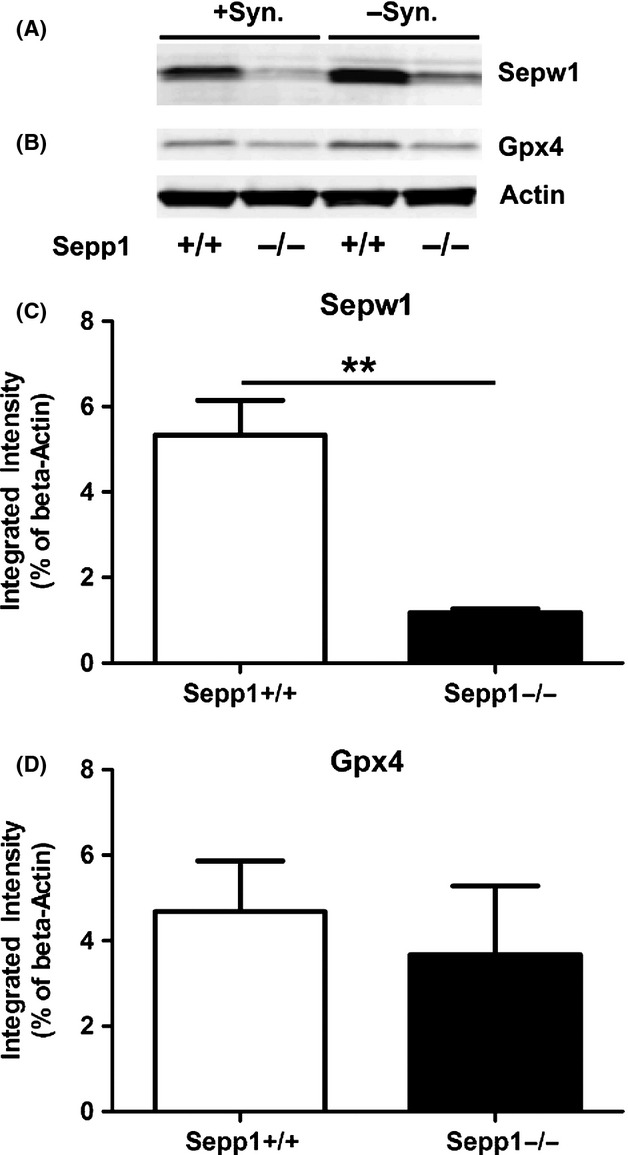
Synaptic expression of selenoprotein W (Sepw1) is reduced in mice lacking selenoprotein P (Sepp1). Synaptosome fractions were prepared from Sepp1−/− and Sepp1+/+ littermate mice. Synaptosome fractions (+Syn.) were analyzed in comparison to S1 fractions (−Syn.). Western blotting for Sepw1 (A) and Gpx4 (B) revealed the presence of both selenoproteins, with beta-actin used as a loading control. Quantitation of synaptosomes revealed that Sepw1 expression (C) was significantly decreased (**P < 0.01) in Sepp1−/− mice (*n* = 3) compared with Sepp1+/+ littermates (*n* = 4). However, Gpx4 expression in synaptosomes (D) was not significantly different between Sepp1+/+ and Sepp1−/− mice.

To assess if Sepw1 is expressed in synapses, we prepared synaptosomes from adult mice and performed western blotting of the purified samples. As Sepw1 expression is reduced in the brains of Sepp1−/− mice, we sought to determine if synaptically expressed Sepw1 is reduced in Sepp1−/− mice compared with wild-type littermate mice. We observed a dramatic decrease in Sepw1 expression in synaptosomes isolated from Sepp1−/− mice compared with control mice (Fig. 4A). Additionally, western blot analysis of Gpx4 showed presence in wild-type synaptosomes, and slightly reduced expression in Sepp1−/− synaptosomes (Fig. 4B). We used beta-actin to control for loading across samples. Quantification of selenoprotein expression in synaptosomes revealed that Sepw1 was significantly reduced to ∼22% of wild-type levels (*t*(5) = 4.309, *P* = 0.0076) in Sepp1−/− mice (Fig. 4C). GPX4 appeared to be reduced in both fractions in Sepp1−/− compared with Sepp1+/+ mice, but the decrease in synaptosomes was not statistically significant (Fig. 4D). These findings indicate that Sepw1 is expressed at synapses, and that Sepp1 is necessary to maintain synaptic Sepw1 expression.

**Figure 5:**
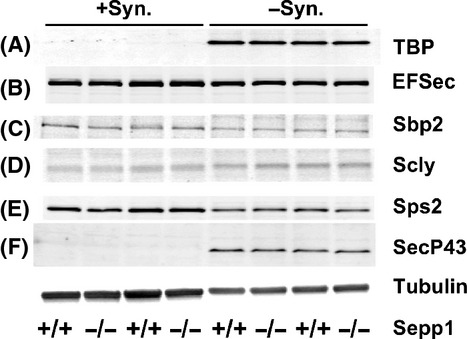
Several selenoprotein synthesis factors are present in synaptosomes. (A) To check for contaminating nuclear proteins, TBP was analyzed. TBP was clearly present in the S1 fractions (−Syn.) but not in the synaptosome fractions (+Syn.). Both EFSec (B) and Sbp2 (C), which are required for insertion of Sec during selenoprotein translation, were present in synaptic fractions. Scly (D), which recycles Se from Sec, and Sps2 (E), which generates selenophosphate for Sec biosynthesis, were both found in synaptic fractions. (F) SecP43, a protein associated with the Sec-specific tRNA, was found only in S1 fractions.

Sepw1 mRNA has been detected in axons, dendrites, and neuropil, in addition to the neuronal somata, suggesting that it may be locally translated in neuronal processes (Willis et al. 2007; Taylor et al. 2009; Cajigas et al. 2012). However, selenoprotein synthesis is unique, and requires several additional protein factors beyond the standard translation machinery. To assess if translation of selenoproteins might occur in distal processes of neurons, we did western blotting of synaptosomes for several proteins involved in selenoprotein translation. Selenoprotein synthesis factors are found in both cytoplasmic and nuclear protein complexes, so we confirmed absence of nuclear contamination by analyzing TATA-binding protein (TBP) (Fig. 5A). Both the Sec-specific elongation factor (EFSec) (Fig. 5B) and the SECIS-binding protein 2 (Sbp2) (Fig. 5C) are required for selenoprotein translation, and were found in synaptosomes. Selenocysteine lyase (Scly) and the Sec-tRNA associated-protein SecP43 are implicated in selenoprotein translation efficiency, but are not a necessary component of the protein translation complex (Squires and Berry 2008; Kurokawa et al. 2011). Scly was detectable in synaptosomes (Fig. 5D), but SecP43 was not (Fig. 5F). Selenophosphate synthetase 2 (Sps2), which produces the Se-donor selenophosphate for selenoprotein translation, was detected in synaptosomes (Fig. 5E). Unlike Sepw1, none of the proteins involved in selenoprotein synthesis were altered in Sepp1−/− mice compared with wild-type controls. This is unexpected for Sps2, which is also a selenoprotein and thus predicted to be dependent on Sepp1 to supply Se.

**Figure 6:**
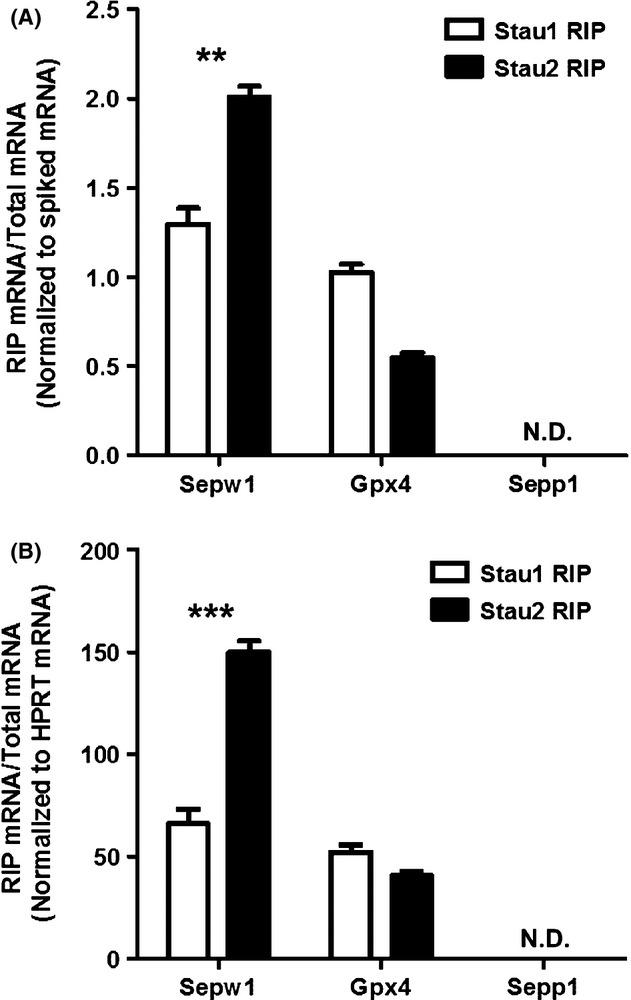
Selenoprotein W (Sepw1) mRNA associates with Stau2 in SH-SY5Y neuroblastoma cells. (A) RT-qPCR was performed on Stau1- and Stau2-RNA immunoprecipitation (RIP) samples and Total RNA samples (*n* = 3) harvested and processed in parallel and normalized to a synthetic mRNA spiked into the samples. Stau2 coimmunoprecipitated more Sepw1 mRNA than the Stau1 immunoprecipitation samples, while also showing greater efficiency compared with Gpx4 mRNA. (B) The qPCR data was normalized to endogenous HPRT mRNA, a putative Stau2-target mRNA. Stau2-RIP coimmunoprecipitates with more Sepw1 mRNA than HPRT mRNA. In contrast, less Gpx4 mRNA is observed in the Stau2-RIP samples when compared with HPRT mRNA. selenoprotein P (Sepp1) mRNA is undetectable in the RIP samples. **P < 0.005, ***P < 0.001.

To uncover a potential mechanism for translational regulation of Sepw1, we performed RNA immunoprecipitation (RIP) experiments using human SH-SY5Y neuroblastoma cells. We immunoprecipitated using antibodies directed at the two paralogs of the RNA-binding protein Staufen, Stau1, and Stau2, which are involved in mRNA transport and localization in neurons (Duchaine et al. 2002). After normalizing to a synthetic RNA spiked into the samples, ∼2% of Sepw1 mRNA was identified in the Stau2-containing mRNP relative to total RNA (Fig. 6A, left). This amount corresponds to a significant ∼1.5-fold enrichment compared with the Stau1-mRNP (*t*(4) = 6.701, *P* = 0.0026). Conversely Gpx4 mRNA had the opposite profile, with more mRNA found in the Stau1-containing mRNP, corresponding to ∼1% of the total Gpx4 mRNA (Fig. 6A, center). Sepp1 mRNA was undetectable in the RIP samples, despite detection in total RNA (Fig. 6A and B, right). We also normalized the data to endogenous HPRT mRNA, which is a putative target of Stau2 in rat brain (Maher-Laporte and DesGroseillers 2010). Sepw1 mRNA associates with Stau2 ∼150% more than HPRT mRNA, while associating with Stau1 ∼33% less than HPRT mRNA (*t*(4) = 9.389, *P* = 0.0007) (Fig. 6B, left). Both Stau proteins associate with Gpx4 mRNA at about 50% or less than their association with HPRT mRNA (Fig. 6B, center). Together, these data argue that Sepw1 mRNA is a specific target of Stau2 in SH-SY5Y cells, and that Stau2-mediated translational regulation of Sepw1 may occur in neurons.

## Discussion

The results reported herein describe the first characterization of regional Sepw1 localization in mouse brain, as well as selenoprotein and synthesis factor expression in synaptosome preparations. Sepw1 is abundantly expressed in neuronal somata and neuropil, and is expressed along with several selenoprotein synthesis proteins in synaptosome fractions. In brains of mice lacking Sepp1, Sepw1 expression is drastically reduced without effect on synthesis factors. Sepw1 mRNA is associated with the RNA-transport protein Staufen 2 (Stau2), further suggesting that Sepw1 can be expressed synaptically.

Selenium is a trace micronutrient that is incorporated into the unique amino acid, selenocysteine (Sec). Sec-containing selenoproteins are typically oxidoreductase enzymes that play crucial roles in reducing reactive oxygen species and oxidized macromolecules. A selenoprotein that is widely distributed across all domains of life, Sepw1, is particularly abundant in brain and muscle of mammals (Gu et al. 2000). Sepw1 mRNA expression is observed in cephalic neural folds and somites in developing rodents, with continued high expression as they become the adult brain and skeletal muscles (Loflin et al. 2006). Sepw1 was initially identified due to its absence in muscle of myopathic Se-deficient lambs, but brain expression of Sepw1, unlike in muscle, is not depleted by dietary Se deficiency (Whanger 2001). However, Sepw1 mRNA and protein expression is reduced in the brains of Sepp1−/− mice (Hoffmann et al. 2007), and the hippocampus is particularly dependent on Sepp1 for maintaining Se (Nakayama et al. 2007). Additionally, Sec lyase (Scly) knockout mice fed a Se-deficient diet display reduced Sepw1 protein in brain compared with wild-type mice (Raman et al. 2012). Therefore, stable expression of Sepw1 in brain during dietary Se deficiency in mice likely depends on both Sepp1 and Scly.

Sepw1 is the smallest described mammalian selenoprotein at ∼10 kDa, and contains an N-terminal thioredoxin-like Cys-X-X-Sec redox motif, where X is any amino acid (Lobanov et al. 2009). As with all selenoproteins, the Sec residue is encoded by a UGA codon in the mRNA. A SECIS in the 3′UTR of the mRNA, the SECIS-binding protein SBP2, and the EFSec help to bypass translation termination and incorporate Sec during translation (reviewed in Squires and Berry 2008). Sepw1 also has another conserved Cys residue in the N-terminal region that is known to bind glutathione (GSH) (Beilstein et al. 1996; Gu et al. 1999). Antioxidant function attributed to Sepw1 is GSH dependent. In vitro experimental studies, which increased or decreased Sepw1 expression, have demonstrated elevated and reduced resistance to oxidizing agents, respectively, but only in the presence of reduced GSH (Jeong et al. 2002). However, siRNA knockdown of Sepw1 increased GPX, superoxide dismutase, and catalase activities, and total antioxidative capability and GSH level in cultured muscle cells, thereby preventing oxidant-induced apoptosis (Xiao-Long et al. 2010). These authors suggested a role for Sepw1 in the antioxidative system that is not direct peroxide detoxification. Thus, the in situ enzymatic role of Sepw1 has remained elusive. Sepw1 mRNA rapidly declines in response to peroxide, suggesting that it has a role in oxidative metabolism. Similar to the metabolic enzyme glyceraldehyde phosphate dehydrogenase (GAPDH), oxidative inactivation of Sepw1 may be involved in rerouting carbohydrate flux from glycolysis to the pentose phosphate pathway, stimulating NADPH generation and reducing the intracellular pool of GSH (Loflin et al. 2006).

The neuroanatomical distribution of Sepw1 may give some insight into its function. Sepw1 is located in dendrites of cortical and hippocampal pyramidal cells as well as cerebellar Purkinje cells. These are large neurons requiring a high rate of energy metabolism and thus may be subject to high oxidative conditions. The location of Sepw1 in hippocampus, cerebellum, and barrel cortex is also coincident with areas highly studied in synaptic plasticity (Lynch 2004; Malenka and Bear 2004). Oxidative stress can increase or decrease synaptic plasticity depending on oxidation levels (Serrano and Klann 2004). Thus, Sepw1, by maintaining redox homeostasis in these regions, may be important for proper synaptic adaptation and development.

A coimmunoprecipitation experiment indicated that Sepw1 interacts with the cytoskeletal microtubule protein tubulin (Dikiy et al. 2007). Our data show some colocalization of Sepw1 with the neuron-specific beta-tubulin, Tuj1. Sepw1 was additionally shown to immunoprecipitate specifically with the beta and gamma isoforms of the 14-3-3 family of scaffolding proteins (Aachmann et al. 2007). A computational study explored a putative reaction mechanism, whereby Sepw1 regulates the oxidation state of a conserved and solvent exposed Cys residue of 14-3-3 beta and gamma (Musiani et al. 2011). Sepw1 was suggested to reduce the oxidized Cys-Sulfenic acid of 14-3-3 back to its parental thiol using the Cys-X-X-Sec motif in combination with the bound GSH moiety. 14-3-3 proteins are abundant in the brain and coordinate the interaction of kinases and phosphatases with receptor and structural proteins, thereby regulating phosphorylation-dependent cellular processes (Berg et al. 2003). The beta and gamma isoforms of 14-3-3 are associated with synaptic vesicle membranes and synaptosomes, with the gamma isoform potentially binding to the synaptic plasma membrane (Martin et al. 1994). Further, 14-3-3 gamma localizes to the vertebrate neuromuscular junction on the postsynaptic side (Strochlic et al. 2004). Like Sepw1 gene expression, the 14-3-3 gamma gene (YWHAG) is highly expressed in brain, skeletal muscle, and heart in humans (Horie et al. 1999).

Sepw1 has been implicated in regulating growth factor-stimulated control of cell cycle-entry in epithelial cells. Knockdown of Sepw1 by siRNA in breast and prostate epithelial cells inhibits EGF-stimulated G1/S transition via nuclear accumulation of p53, leading to induction of p21 and G1 arrest (Hawkes and Alkan 2011; Hawkes et al. 2012). Cell cycle arrest in this context was mediated by MKK4 and downstream MAPK signaling (Hawkes and Alkan 2012). Additionally, recovery from G2 arrest caused by DNA damage was reduced by siRNA knockdown of Sepw1, via dissociation of CDC25B from 14-3-3 (Park et al. 2012). 14-3-3 proteins regulate phosphorylation-mediated cell signaling including MAPK pathways; thus, Sepw1 may function in signal transduction from receptors to target proteins via reactive oxygen intermediates.

High muscle expression of Sepw1 mRNA is associated with myoblasts, and expression is decreased in differentiated myotubes (Loflin et al. 2006). Thus, the abundance of Sepw1 mRNA and protein in postmitotic neurons is mysterious. Sepw1 mRNA is widely expressed in neurons, including apparent expression in axonal and dendritic compartments [(Willis et al. 2005, 2007; Taylor et al. 2009; Cajigas et al. 2012) supplemental data]. Whether translation of Sepw1 occurs in these distal cellular compartments is uncertain. Selenoprotein translation in mammals specifically requires the proteins Sbp2 and EFSec, in addition to the standard translation machinery. Both of these proteins were identified in synaptosomes along with Sps2 and Scly, which are important in Sec metabolism. The major protein involved in selenoprotein translation that was not investigated in this study is the Sec-synthetase enzyme, SepSecS. SepSecS, also known as soluble liver antigen/liver pancreas antigen, is required to generate the Sec-loaded tRNASec (Palioura et al. 2010). We were unable to test for the presence of SepSecS in synaptosomes. However, given the proteins identified in synaptosomes, synthesis of Sepw1 at or near synapses appears to be plausible.

Selenoprotein mRNAs are thought to be packaged into mRNP complexes, which aid in preventing nonsense codon-mediated decay (NMD) of transcripts with a Sec-specifying UGA that could be interpreted as a premature termination codon. Staufen proteins Stau1 and Stau2 are involved in a related process termed Staufen-mediated decay (Park et al. 2013), and we have shown that Sepw1 mRNA associates with Stau2 in SH-SY5Y cells. This finding is supported by data showing that Sepw1 mRNA is found in Stau2-mRNP complexes in both HEK293 cells and embryonic rat brains ([Maher-Laporte and DesGroseillers 2010] supplemental data). Stau2 is particularly abundant in brain and contributes to dendritic transcript localization and translational regulation (Duchaine et al. 2002; Mikl et al. 2011). Here, we demonstrate that Sepw1 is highly expressed in brain and synapses, and suggest that its translation is under control of RNA-binding proteins such as Stau2. In addition to Stau2, DJ-1/Park7 has been experimentally demonstrated to coimmunoprecipitate with Sepw1 mRNA in M17 human neuroblastoma cells and human brain tissue (van der Brug et al. 2008; Blackinton et al. 2009). DJ-1 is a multifunctional redox-sensitive protein that is associated with Parkinson's disease, stroke, and cancer (Kahle et al. 2009). DJ-1 protein has shown varying degrees of localization to synapses, axons, and dendrites (Olzmann et al. 2007; Usami et al. 2011), further suggesting the local regulation of Sepw1 expression in distal neuronal compartments. These proteins and additional mechanisms may ultimately provide specific and sensitive spatiotemporal control of Sepw1 expression in neurons.

In sum, we have shown that Sepw1 expression is abundant in several mouse brain regions, including pyramidal neurons of cortex and hippocampus, and Purkinje cells of cerebellum. We also showed Sepw1 expression in neuronal processes, especially apical dendrites, and some colocalization with tubulin. Analysis of isolated nerve terminals further revealed the presence of Sepw1 and much of the selenoprotein synthesis machinery in synaptic compartments. Sepw1 expression was drastically reduced in hippocampus and synaptosomes in Sepp1−/− mice. Lastly, Sepw1 mRNA associates with Stau2 in SH-SY5Y neuroblastoma cells. Combined with previous reports documenting Sepw1 mRNA expression in neuronal processes, translational regulation of Sepw1 expression in synaptic compartments is probable and warrants further investigation.
